# Editorial: Methods in thoracic oncology

**DOI:** 10.3389/fonc.2023.1178236

**Published:** 2023-05-10

**Authors:** Jelena Stojšić, Tatjana Adžić-Vukičević

**Affiliations:** ^1^ Service of Pathology, University Clinical Centre, Belgrade, Serbia; ^2^ School of Medicine, University of Belgrade, Belgrade, Serbia

**Keywords:** methodology, oncology, thorax, small cell lung cancer, non-small lung cancer

Lung carcinoma is the most common tumor of the thoracic region and therefore requires a serious multidisciplinary approach in diagnostics, treatment, and in determining the prognosis and outcomes of each specific case, using biochemical, radiologic, and pathohistological methods and parameters. A contemporary multidisciplinary approach is also adopted for other tumors of the thoracic region. In the last two decades, for thoracic tumors, and especially lung carcinoma, various predictive markers that influence treatment options and thus also the outcome of diseases and the quality of life of patients have been developed.

All accepted and published papers have very studiously examined possibilities for establishing new methods, i.e. new prognostic parameters in thoracic oncology. Since the studies included large numbers of patients, we may consider their results relevant. Topics covered by these studies ranged from biochemical and metabolic properties to the microenvironment of malignant cells of both types of primary lung carcinoma, as well as secondary, metastatic lung carcinoma. Some studies also presented rare thoracic and lung tumors and the ways they were diagnosed, underlining the importance of pathologic examinations in their diagnostics. The contribution of imaging tools to the prognostics of malignant diseases in thoracic oncology was also emphasized, particularly in correlation with the metabolic changes observed. One of the ways of studying the introduction of new methods was by using experimental animal models.

The studies covered all common histological types of lung carcinoma, as well as the most common secondary or metastatic intestinal or colorectal carcinoma and chest wall tumors. A rare variant of the most aggressive papillary subtype of lung adenocarcinoma of size ≤20mm (pTNM: T1-2N0M0) was also presented in this Research Topic. The rare minute pulmonary meningothelial-like nodules were described, and one of the articles considered their specific clinical, radiological, morphological, and immunohistological features. The article also described a cytological investigation for the purpose of differentiating malignant from benign cells in pericardial effusion. This cytological study gained validation of the scoring system for their differentiation, which is a challenge in daily practice. An original experimental investigation of malignant airway stenosis in rabbits was for the first time published in the global literature. Two studies investigated biochemical and metabolic mechanisms of remodeling the microenvironment in NSCLC and SCLC progression and metastasis.

The details of each of these articles are the following:

The new predicting method in the evaluation of staging of SCLC combines the role of PET/CT metabolic parameters, systemic immune-inflammation marker (SII), maximum standardized uptake value (SUVmax), metabolic tumor volume (MTV), total lesion glycolysis (TLG), systemic immune-inflammation marker (SII), maximum standardized uptake value (SUVmax), metabolic tumor volume (MTV), and total lesion glycolysis (TLG) with inflammatory markers, namely, neutrophil/lymphocyte ratio (NLR), platelet/lymphocyte (PLR), and monocyte/lymphocyte ratio (MLR). The binary stage system, extensive-stage disease (ED) and limited-stage disease, was included in 119 patients with previous pathological diagnoses of SCLC who had performed PET/CT scans. The authors detected that baseline markers and tumor metabolic parameters were associated with a binary stage in SCLC patients. ED-SCLC could be predicted on PET/CT scans in patients with associated high levels of MTV/MLR based on metabolic tumor volume and systemic inflammatory response (Hu et al.).

In another multidisciplinary and multicentric study researchers tried to find a correlation between immune cells and high-density lipoprotein as the markers for early detection of metastatic NSCLC. The authors investigated the value of blood-related indicators: neutrophil/leukocyte (NLR), lymphocyte/monocyte (LMR), high-density lipoprotein (HNR), high-density lipoprotein/monocyte (HMR), and combined assays in metastatic NSCLC. The researchers concluded that NLR, LMR, HNR, and HMR levels had diagnostic values for metastatic NSCLC. This investigation provides a mechanism for remodeling the microenvironment prior to NSCLC metastasis. The idea is to continue the research in the future, including more patients with metastatic NSCLC for better evaluation of investigated markers and adopt it as a new method (Zhang et al.).

Two studies investigated the performance of the imaging method of 3D deep learning that automatically predicts tumor invasiveness in intraoperative frozen tissue sections, comparing it with the results of an interobserver study between radiologists on high-resolution CT scan (HRCT) and surgeons in conditions where pathologists established diagnosis of low-risk adenocarcinoma (pre-IAC), adenocarcinoma *in situ* (AIS), or minimally invasive adenocarcinoma (MIA). The deep learning approach will be a valuable guiding strategy during surgery (Lv et al.). In the second study, the deep learning method was established as a recommended prediction model for the detection of sub-solid tumor nodule growth pattern, mass, and measures, intended to successfully manage them during follow-up periods, based on approximately 2,500 investigated lung tumors (Liao et al.).

The group of authors tried to provide the development and validation of a nomogram for predicting individual survival (OS) and lung cancer-specific survival (LCSS) prognostic model for the early-stage T1-2N0M0 subset of SCLC. A retrospective population-based study included approximately 1,600 patients with SCLC from the SEER database, divided into two cohorts depending on the year of diagnosis. This investigation provided a new nomogram showing certain reliability that could aid clinicians in improving the prognosis, treatment strategy, and new design of future clinical trials suitable for web servers as well. (Ge et al.).

Chest wall tumors, including metastatic lung, breast and thymic carcinoma, mesothelioma, and sarcoma used to be surgically treated. The authors of a retrospective, multicentric study strongly suggested salvage brachytherapy (SABT) as a safe and efficient method. This type of therapy shows promising efficient follow-up in patients with Karnofsky scores higher than 80 (ECOG 0 and 1), who received a dose greater than 130Gy and had tumors larger than 40mm (Huo et al.).

Pulmonary adenocarcinoma rarely has a micropapillary (MP) pattern. Authors investigated preoperatively diagnosed MPs ≤20mm (pTNM: T1-2N0M0) in 390 patients in different proportions. The prognosis depends on the type of resection and lymph node dissection. Lobectomy and systemic lymph node dissection are recommended for patients with a micropapillary histological component of >5% and sublobular resection and limited lymph node dissection are recommended for patients with a micropapillary histological component of ≤5%. The authors will evaluate the feasibility of this method in the future (Xu et al.).

Metastatic intestinal adenocarcinoma or colorectal carcinoma (CRC) in lung parenchyma was investigated in patients meeting established criteria. The leading criterion was three or more metastatic nodules in the lung parenchyma in this trial. The conclusion could be that new biomarkers are needed for better risk stratification and identification of patients with a high risk for CRC recurrence after metastasectomy without conventional markers. Circulating DNA (ctDNA) will be analyzed at various pre-and post-surgical time points as well as in surgically untreated patients to characterize its role as a clinically useful biomarker in patients with CRC undergoing pulmonary mastectomy. This trial will provide stronger evidence for the performance of pulmonary metastasectomy and potentially better patient selection (Schmid et al.).

The etiology of pericardial effusion was emphasized as very significant in patients with diagnosed tumors in the surrounding tissues. The authors suggested a scoring system for differentiating malignant from benign cells in pericardial effusion. Included parameters are loss of weight (3 points), no fever (4 points), and mediastinal lymph node enlargement (6 points). In pericardial effusion, the parameters are the presence of adenosine deaminase (5 points), effusion lactate dehydrogenase (7 points), and carcinoembryonic antigen (10 points). The cut point is 16 for differentiating malignant from benign cells. The reviewers remark that pericardial effusion pathology can resolve the dilemma with cell morphology and immunohistochemistry as an updated model (Jin et al.).

The rabbit model is available for stent implantation in case of tumors growing in the bronchial tree, particularly in the trachea. This method is safe and effective for stent implantation due to it facilitating the treatment approach. The author recommends this model for preclinical animal studies on bronchoscopic interventional treatments (Wang et al.).

In a multidisciplinary approach, combining clinical, radiological, morphological pattern, and immunohistochemical findings, seven cases of minute pulmonary meningothelial-like nodules (MPMNs) were diagnosed and described, incidentally detected with lung carcinoma and pneumonia, and in three cases occurred alone, with the appearance of multiple, medium hardness, and greyish-white solid tumors. A typical growing pattern through widened alveolar septa, morphology, and immunoprofile confirmed MPMNs (Wang et al.).

All published articles could be summarized as novelties in multidisciplinary methods in thoracic oncology. All of them are interesting and attractive for readers, from pulmonologists and thoracic surgeons to thoracic pathologists and oncologists. We expect these studies to inspire researchers to recommend and adopt new methods that might contribute to diagnostics and help determine prognostic and predictive methods in thoracic oncology.

## Author contributions

This is the editorial of accepted papers that will be published in the journal with specific topic “Editorial: Methods in Thoracic Oncology”. This paper is the summary of all accepted papers where the new methodology in thoracic oncology is suggeted or old methods that are improved. As editori, I analyzed all accepted papers and presented a new methodology and contributions, i.e. obtained results introducing a new methodology. The co-author helped with clinical experiences interpreting obtained result in eleven accepted papers. All authors contributed to the article and approved the submitted version.

